# Advances in Fine Needle Aspiration Cytology for the Diagnosis of Pulmonary Carcinoma

**DOI:** 10.4061/2011/897292

**Published:** 2011-06-27

**Authors:** Adnan Hasanovic, Natasha Rekhtman, Carlie S. Sigel, Andre L. Moreira

**Affiliations:** Department of Pathology, Memorial Sloan-Kettering Cancer Center, 1275 York Avenue, New York, NY 10065, USA

## Abstract

New developments in the field of thoracic oncology have challenged the way pathologists approach the diagnosis of pulmonary carcinoma. Nonsmall cell carcinoma is no longer an adequate diagnostic category. Pathologists are required to further classify tumors into adenocarcinoma and squamous cell carcinoma since specific therapies are now recommended depending on the histological tumor type. This change occurred following the discovery of specific molecular alterations that predict response to certain drugs and now molecular testing of tumor cells is often requested to direct therapy. The vast majority of lung cancer is diagnosed in advanced clinical stages, where cytologic or small biopsy material is the only form of tissue diagnosis, thus placing cytology, especially fine needle aspiration biopsy in the front line for management of patients with lung cancer. In this paper we will review the current concepts in the suitability and accuracy of fine needle aspiration biopsy, including diagnosis, classification, prognostic markers, and use of ancillary techniques.

## 1. Introduction

Pulmonary nodules discovered by an imaging technique present a relatively frequent clinical problem. A solitary pulmonary nodule is a common manifestation of a benign condition. However, in nodules larger than 2 cm, the incidence of a primary lung cancer ranges from 64 to 82% [1]. An early, accurate diagnosis is of paramount importance for initiating specific therapy for malignant lesions, and for avoiding unnecessary procedures for benign conditions. Thus, after clinical risk assessment tissue diagnosis is the next step in managing radiologically suspicious lung nodules. Direct tissue sampling for diagnosis is essential in most patients for decisions regarding treatment and can be accomplished by fine needle aspiration biopsy (FNAB), endoscopic or core needle biopsy, or surgical resection. Sampling of the lesion by FNAB can be performed via the airway (endobronchial transbronchial FNAB) or chest wall (CT-guided percutaneous FNAB). Transbronchial FNAB is useful for the diagnosis of primary pulmonary lesions that lie beneath the bronchial surface and for staging lung cancer patients by sampling mediastinal lymph nodes. FNAB has become recognized as a safe and effective diagnostic tool, as a result of improved aspiration biopsy tools and techniques, better control of complications, and increased experience of cytopathologists in interpreting aspirate specimens.

Most patients with lung cancer present with clinical advanced disease and therefore are not candidates for surgery with curative intent, but are rather treated with systemic therapies. In the age of personalized therapies, cytological material in the form of FNAB may be the only available diagnostic specimen, and the only material available for molecular studies, necessary for current therapeutic decision making. New recommendations for screening of high-risk populations [2] coupled with the ongoing development of minimally invasive techniques and procedures for sampling lung lesions will most likely further increase the need for accurate diagnosis and molecular characterization of malignant tumors on small biopsy specimens.

In this paper, we will cover current concepts and advances in FNAB of pulmonary carcinomas including diagnosis, classification, prognostic makers, and use of ancillary techniques.

## 2. Clinical Advances in the Management of Patients with Pulmonary Carcinoma

The current classification of lung cancer recognizes four major histological subtypes, namely, squamous cell carcinoma (SQC), adenocarcinoma (ADC), large-cell carcinoma (LCC), and small cell lung carcinoma (SCLC). Until recently, most of the cytological diagnosis of lung carcinoma was based on distinguishing SCLC from other tumors generally designated as nonsmall cell carcinoma (NSCLC), because these two categories were the most relevant for directing therapy. However, advances in thoracic medical oncology have led to a paradigm shift in NSCLC diagnosis, resulting in a new emphasis on accurate NSCLC subtyping. Specifically, two novel agents have challenged NSCLC as clinically relevant diagnostic category. It has been demonstrated that patients with the diagnosis of SQC are at increased risk for life-threatening complications if treated with bevacizumab, a humanized antibody against vascular endothelial growth factor (VEGF) [3]. In addition, in the case of pemetrexed, an antifolate that inhibits multiple enzymes in purine and pyrimidine synthesis, patients with SQC showed no response to the drug in comparison to a good response observed in patients with the diagnosis of non-squamous cell carcinoma [4]. For these reasons, these two new drugs are only recommended for use in patients with a diagnosis of non-squamous cell carcinoma. Other developments include the identification of genetic alterations which have been described almost exclusively in adenocarcinoma that confer susceptibility to therapeutic agents or resistance to chemotherapeutic drugs. For example, tumors with epidermal growth factor (*EGFR*) mutations have a better outcome and respond to the tyrosine kinase inhibitors erlotinib and gefitinib, as a first-line therapy, whereas patients without *EGFR* mutations seem to have a better outcome with standard chemotherapy [5]. Furthermore, translocation in the *EML4-ALK* gene has been described predominantly in adenocarcinomas. This translocation confers susceptibility to specific inhibitor, crizotinib that is currently undergoing clinical testing [6]. 

These advances in the understanding of molecular mechanisms underlying lung cancer and the development of new targeted therapies challenge the traditional diagnostic dichotomization between SCLC and NSCLC and prompt a more specific characterization of NSCLC into squamous or adenocarcinoma category. Traditionally, NSCLC subclassification has been based on morphologic assessment of routine H&E-stained histological specimens. Because cytology specimens, such as FNAB, differ in preparation and technique from traditional histology, the accuracy of subtyping these specimens has been challenged, yet there is considerable evidence supporting the utility of cytology in both subtyping NSCLC and providing material for predictive and prognostic studies.

## 3. Role of Immediate Assessment in the Accuracy of Lung FNA Cytological Diagnosis

Published reports reveal that the sensitivity of FNAB for the diagnosis of lung cancer ranges from 56 to over 90% whereas specificity is close to 100%. In nearly all these studies, the overall positive predictive value is nearly 99%. While the false positive rate is generally less than 1%, a negative result is less reliable with most studies reporting a false negative rate of around 10% [7, 8]. The major contribution to the relatively high false negative rate is failure to obtain diagnostic material, most commonly due to sampling error. Studies have shown that immediate on-site assessment is valuable in minimizing false negative diagnoses due to nondiagnostic material [9, 10]. Published series by Austin and Cohen show that immediate on-site assessment during FNAB was associated with a statistically significant increased diagnostic accuracy compared to cases without immediate assessment, 100% versus 80%, respectively [11]. During on site adequacy determination, smears from the aspirate are rapidly stained and are evaluated by a cytopathologist or cytotechnologist for cellularity and diagnostic yield. On-site adequacy evaluation also provides real-time communication of information including appropriate tissue triage recommendations for ancillary tests such as molecular testing, flow cytometry, cytogenetics, electron microscopy, and so forth. This interaction directly impacts clinical management during the critical diagnostic phase while the lesion can still be readily sampled.

## 4. Accurate Morphological Diagnosis of Subtypes of NSCLC

Morphology still remains the cornerstone in lung cancer classification. The World Health Organization classification of lung tumors through the 1999 edition did not address lung cancer diagnosis based on small biopsies and cytology [12]. In the 2004 World Health Organization classification, cytology was addressed for the first time, with descriptions of the morphological criteria for each type of pulmonary carcinoma [13]. In the new revised proposal [14], an entire section is dedicated to the classification of lung tumors based on small biopsy material including FNAB. This highlights the importance and recognition of the role that FNAB plays in the diagnosis and management of pulmonary carcinomas. 

Lung cancer histological subtypes that are morphologically recognizable on cytology specimens are ADC, SQC, and SCLC, as well as carcinoid tumors. Other types of lung carcinoma such as large cell carcinoma and other rare variant as fetal type and colloid adenocarcinoma may be suspected on the basis of pure morphology but usually require evaluation of the surgically resected specimen for the final diagnosis. 

Historically, it has been important to accurately identify SCLC as the treatment is different from NSCLC. Classical morphological features of SCLC such as nuclear molding, frequent mitoses, and absence of nucleoli are often distorted on a small biopsy specimen showing extensive crush artifact. In this setting, cytology has an edge over histology because of better preservation and fewer artifacts [15]. Cytology is highly accurate and a well-recognized method to distinguish SCLC from NSCLC. In a study of 259 consecutive lung FNAs by Delgado et al., SCLC was distinguished from NSCLC with accuracy of 96% [16].

**Figure 1:**
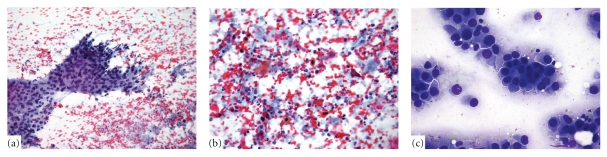
The characteristic morphologic patterns of tumor in smears. (a) SQC carcinoma showing flat sheet of polygonal, atypical cells and (b) orangeophilia on Pap stain demonstrating keratinization. (c) ADC with typical cytomorphology and formation of glandular structures.

Unlike the distinction of SCLC and NSCLC, feasibility of NSCLC subtyping in cytology has been controversial. However, based on a recent study from our institution, cytology provides several advantages over surgical specimens for the subtyping of NSCLC [17]. The key morphologic criteria for ADC versus SQC are glandular architecture versus keratinization, respectively. The Papanicolaou (Pap) stain has exquisite sensitivity for even minimal keratinization aiding in the distinction of SQC from ADC. The morphologic patterns which emerge in tumor smears provide a clue to a tumor subtype which may not be apparent in surgical specimens (Figure 1). In addition, due to immediate fixation, cytology provides greater nuclear and cytoplasmic resolution than histology. While in the majority of cases a line of differentiation can be clearly identified by morphology, difficulties arise in a subset of cases. 

In a recent study, which included 165 cases with paired FNAB and resection diagnosis of ADC and SQC, we described some of the limiting factors for the interpretation and accurate classification in cytology specimens. The strongest predictors for difficulty in subtyping were poor differentiation of the tumor where distinguishing morphologic features are not apparent (Table 1), followed by scant cellularity. Nonkeratinizing poorly differentiated squamous cell carcinoma in particular is subject to misclassification by FNAB [18]. Another difficulty is presented by tumors with mixed histology, but true adenosquamous carcinomas are infrequent with reported incidence of 2 to 3% in published surgical series [19]. 

Despite these limitations, using morphology and occasional immunocytochemistry we observed that when faced with the need to subclassify NSCLC we performed with high concordance between cytology and resected specimens of 97% and 93% for identifying squamous and adenocarcinoma, respectively (Table 2). Despite the lower sensitivity for non-keratinizing SQC, the specificity of this diagnosis is very high, which means that the false-positive classification as SQC is extremely rare. Thus having proved overall high accuracy of cytology in distinguishing SQC versus non-SQC it was concluded that cytological specimens are suitable for guiding therapeutic decisions within these diagnostic categories [18].

## 5. Use of Immunohistochemistry Stains in the Characterization of NSCLC Subtypes

**Figure 2:**
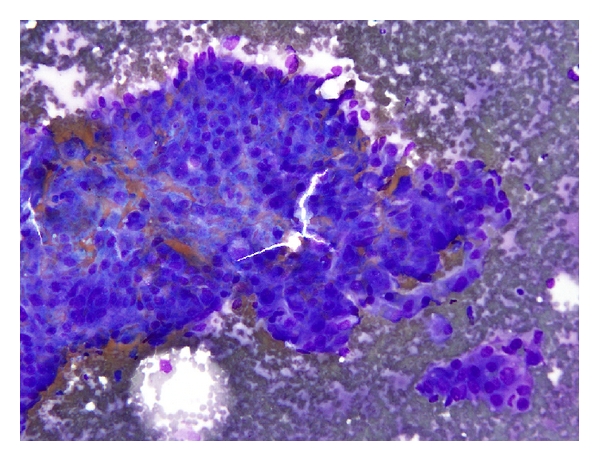
Poorly differentiated carcinoma lacking clear signs of differentiation on light microscopic examination. Further work up using limited panel of antibodies will differentiate tumor cell lineage in the majority of cases.

In tumors that do not show clear-cut signs of differentiation on light microscopy examination (Figure 2) further investigation by immunohistochemistry may highlight tumor cell lineage. In biopsies like FNAB, with limited material, the need to conserve material for possible mutational analysis and other prognostic markers obligates the use of the most efficient and limited panel of immunohistochemical stains. Several recent publications have addressed the question of what is the best panel of markers to be used in the distinction between adenocarcinomas and squamous cell carcinoma. Results published by Wu et al. showed the advantages of immunocytochemistry in distinguishing poorly differentiated SQC from SCLC of the lung, and primary adenocarcinoma from metastatic tumors in cytology specimens. In their study, all poorly differentiated squamous cell carcinomas showed expression of p63 while negative for thyroid transcription factor-1 (TTF-1), whereas adenocarcinomas had the opposite staining pattern. Of note, SCLC had identical p63/TTF-1 expression profile as adenocarcinoma, but application of standard morphologic criteria and addition of neuroendocrine markers was sufficient for accurate classification [20]. Nicholson et al., in their recently published work that included 13 FNA cytology specimens, showed that a limited panel of TTF-1, CK5/6, and p63 together with a mucin stain, refined diagnosis of NSCLC to either ADC or SQC in 65% of cases [21]. 

In summary, most of the work has been concentrated on the expression of 3 markers TTF-1, p63, and a high-molecular-weight cytokeratin (HMWCK). The following algorithm, based on published data and our experience can guide the interpretation of the stains. A TTF-1 negative/p63-positive/HMWCK-positive profile is supportive of SQC, whereas any expression of TTF-1 is supportive of ADC. It is worth mentioning that coexpression of TTF-1 and p63 can be seen in adenocarcinomas. In most cases p63 expression in adenocarcinoma is patchy and weak. Negative staining for both p63 and TTF-1 usually rules out squamous cell carcinoma [22].

Napsin-A is an aspartic proteinase, involved in the maturation of the surfactant protein B and is expressed in the cytoplasm of cells of lung and kidney [23]. The staining is cytoplasmic and is strongly positive in up to 80% of primary lung adenocarcinomas. In the study of Stoll et al., 75 cytology cases were analyzed. It showed that the sensitivity and specificity of TTF-1 were each 81%. Napsin-A exceeded the specificity of TTF-1 at 96% with a lower sensitivity of 65%. In the study, the only carcinoma of nonlung origin in which Napsin-A was detected was renal cell carcinoma, suggesting that Napsin-A can be used as a surrogate marker in work up of poorly differentiated lung adenocarcinoma or an unknown primary tumor [24]. Desmocolin-3, constitutive protein of desmosomes, is found to be overexpressed in SQC of the lung [25, 26]. In the study by Monica et al., which included 31 cytological specimens originally classified as NSCLC, staining for desmocolin-3 and TTF-1 was mutually exclusive in tumors [26].

Several authors reported on the high sensitivity and specificity of miRNA expression in SQC of the lung, and its usefulness in differentiating ADC from SQC in small specimens [27, 28]. However, a recently published study does not support this observation [29]. The utility of these new approaches compared to standard markers needs to be evaluated further and validated, since a limited panel of immunohistochemical markers can reproduce the results obtained by the new molecular techniques [22].

## 6. Adequacy of Material Obtained by FNAB for Molecular Testing

In the recent years we have witnessed a revolution in our understanding of the molecular basis of NSCLC. These advances have led to development of multitude of commercially available prognostic and predictive biomarkers and targeted therapeutic agents. Despite these advances in treatment, the overall prognosis remains poor in patients with advanced disease. Personalizing therapy based on an individual tumor molecular profile can optimize efficacy with the available agents. Molecular determinants that guide treatment decision-making may have a prognostic or predictive function, and are commonly referred to as prognostic or predictive markers, respectively. Prognostic marker refers to a tumor characteristic that is useful for estimating a patient's outcome independent of therapeutic decisions. In contrast, predictive markers are useful in making therapeutic decisions. Mutations of *EGFR, KRAS*, and *EML4/ALK* translocations are mutually exclusive in lung ADC and identify tumor subsets with unique dependencies and drug sensitivities. *KRAS *mutation testing is utilized by some institutions to exclude *EGFR* mutations or an *EML4/ALK* translocation. 

In particular *EGFR* mutation and *EML4/ALK* fusion gene testing have reached clinical validation and are incorporated into the current treatment paradigm [30]. *EML4/ALK* testing also appears to have clinical utility in identifying patients who could benefit from referral for a study targeting *ALK* inhibition, such as ongoing phase 3 studies of the small molecule tyrosine kinase inhibitor crizotinib.

There is increasing awareness that the quality of specimens, such as cytology, has a profound influence on molecular diagnostic test results. FNAB samples are exposed to a greater variety of cytopreparation methods than resected tissue and sample size and heterogeneity may have an effect on the downstream molecular test results [31]. Most molecular techniques including in situ hybridization, polymerase chain reaction (PCR), and transcriptional profiling can be done on FNAB specimens [32, 33]. Optimizing and standardizing of FNAB sample preparation methods is needed to preserve biomolecular integrity to enable seamless integration into molecular testing.

At present, there are few studies that rigorously compare the cellular composition of FNA samples with the quantity and quality of the desired analyte (DNA, mRNA, or protein) or the robustness of the biomarker test utilizing the sample. Schuurbiers et al. in their study conclude that molecular testing of *EGFR* and *KRAS* on cytologic material obtained by endobronchial ultrasound-guided transbronchial FNAB is feasible and could be performed on 77% of their specimens [34]. Another study by Smouse et al. showed that 67% of cytology specimens were adequate for molecular testing with some of the samples having as little as 25% tumor cellularity [35]. 

We have found that in a large screen of various cytologic specimens (FNABs, effusions, and exfoliative specimens) submitted for *EGFR* or *KRAS *mutational *testing*, 98% of samples were suitable for analysis. We concluded that testing is feasible and that with rare exception all cell blocks subjectively interpreted as “adequate” for diagnosis by a pathologist yielded sufficient quantity and quality of DNA for mutational analysis. This finding is in agreement with several other studies [18]. In a study of consecutive specimens from our institution, it was found that 79% of cytology specimens and 89% of small biopsy specimens submitted for molecular testing were sufficiently cellular [36]. The rate of *EGFR* and *KRAS* mutations detected in cytologic specimens in the study was comparable to the rate detected in surgical specimens. 

## 7. Use of FNAB for Prognostic Markers

The reported incidence of local or distant recurrence following surgical resection of early-stage NSCLC is around 36% [37]. The risk of recurrence is clearly linked to clinical stage but further biomarkers predictive of tumor recurrence are needed. A histological grading system based on the predominant histological patterns seen in pulmonary adenocarcinoma has been described recently [38]. In that study, patients with stage 1 pulmonary adenocarcinoma could be accurately stratified according to risk of recurrence of death of the disease into 3 tiers representing low, intermediate and high risk, thus indicating that an objective system of tumor grade had prognostic significance. In cytological material however, there was no reliable corresponding pattern of cellular aggregates to predict a histological pattern for the same tumor [39], therefore a grading system based on nuclear features has been developed and evaluated in FNAB of pulmonary adenocarcinoma. The cytology grading system is based on the nuclear size of neoplastic cells; pattern of chromatin distribution; and nuclear contour and it separates stage 1 pulmonary adenocarcinomas into two groups. Tumors with low nuclear scores show a lower risk of recurrence or death of the disease in contrast to tumors with high nuclear scores [40]. In conclusion, FNAB can provide significant prognostic information to clinicians managing patients with pulmonary adenocarcinoma.

*EGFR *mutations are the best predictor of response to *EGFR* kinase inhibitors in pulmonary adenocarcinoma. Recently, two antibodies that detect specifically mutated EGFR proteins have become commercially available. A recent study from our institution demonstrated that these antibodies show sensitivity of 95% for the detection of EGFR L858R mutation, and sensitivity of 85% for the detection of exon 19 deletions. They concluded that immunohistochemistry for mutated EGFR could be used as a screen method to identify candidates for therapy with EGFR tyrosine-kinase inhibitors [41].

An antibody that correlates with *ALK* gene rearrangement in NSCLC has also been reported recently with promising results [42, 43] however this antibody is not yet commercially available.

The use of these antibodies in cytological material has not yet been validated, but they may offer an invaluable tool for screening patients with lung cancer where cytological material is the only specimen available.

## 8. New Sampling Methods in Lung FNAB (EBUS and ENB)

A couple of new minimally invasive procedures have been recently developed as an alternative to standard approaches. One of them, endobronchial ultrasound-guided transbronchial needle aspiration (EBUS-TBNA) has recently emerged as a valid minimally invasive method for mediastinal staging of lung cancers and diagnostic workup of centrally located masses [44]. When used to sample mediastinal lymph nodes, at least a moderate number of lymphocytes must be present to ensure the adequacy of the specimen and avoid a false negative result. A large retrospective study performed at our institution found that EBUS-TBNA had 89% sensitivity and 100% specificity for malignant disease, revealing no major discrepancies between tumor subtypes rendered by EBUS-TBNA cytology and histology [45]. A study by Turnoy et al. showed that in patients with NSCLC without extrathoracic metastasis, EBUS-TBNA reduces the need for surgical staging by 68% with lower incidence of complications and no difference in diagnostic performance, thus establishing the procedure as a valid alternative to mediastinoscopy [46]. 

The two major limitations of standard flexible bronchoscopy are its inability to reach peripheral segments of the lung and the limited diagnostic yield from lesions less than 3 cm in diameter. The alternative to bronchoscopy is CT-guided percutaneous biopsy where the possible complications include hemorrhage and pneumothorax. Recently developed technology that is emerging in clinical practice essentially combines these two methods. Electromagnetic navigation is a localization device that assists in placing endobronchial accessories (e.g., forceps, brush, and needle) in the desired areas of the lung. The system uses low frequency electromagnetic waves and real-time 3D digital reconstruction of the previously obtained CT scan of the bronchial tree. Electromagnetic navigational bronchoscopy (ENB) systems were recently cleared by the US Food and Drug Administration to aid the physician in guiding endoscopic tools in the respiratory tract. This novel technical advance localizes and samples lesions in the lung parenchyma and mediastinum that are beyond the reach of standard endoscopy [47]. Recent published studies showed the diagnostic yield of ENB for small peripheral lung lesions are in the range from 54% to 77%. Lamprecht et al. studied ENB sampling using rapid on site cytological evaluation during the procedure and it showed sensitivity and specificity of 84.6% and 100%, respectively. Citing potential drawbacks, they found that 33.3% of the cases with ENB sampling were falsely negative and definitive diagnosis had to be established by CT-guided biopsy or by surgery [48]. Of note, other authors have reported that presence of cytologist virtually eliminates the problem of inadequate samples [49, 50].

## 9. Conclusion

The field of thoracic oncology is going through a revolution with the advent of targeted therapy for the management of patients with lung cancer. FNAB is in many cases the only diagnostic specimen available for guiding therapeutic decisions. FNAB has proven to be an invaluable tool not only for diagnostic accuracy of pulmonary carcinomas classification, but also as a reliable and adequate source of material suitable for molecular analysis.

## Figures and Tables

**Table 1:** Factors contributing to difficulty of cytologic subtyping of adenocarcinoma and squamous cell carcinoma.

*N* = 165	Cellularity		Differentiation^(1)^		Histologic type	
	Low	High	Well-moderately	Poorly	ADC	SQC
Correct definitive Subtyping (*n* = 148)	82%	94%	87%	69%	93%	70%
Difficulty in Subtyping (*n* = 17)^(2)^	18%	6%	13%	31%	7%	30%

^(1)^Grade of differentiation was based on resected specimens.

^(2)^Difficulty in subtyping encompasses cases which were incorrectly classified, unclassified, or underclassified/subtype favored by cytology.

**Table 2:** Sensitivity and specificity of cytologic tumor subtyping.

*n* = 183	Sensitivity	Specificity	Accuracy
SQC versus non-SQC	87%	98%	97%
ADC versus non-ADC	98%	79%	93%
